# Development of a diagnostic sensor for measuring blood cell concentrations during haemoconcentration

**DOI:** 10.1177/0267659116667806

**Published:** 2016-09-24

**Authors:** Craig A. Robertson, Terence Gourlay

**Affiliations:** Department of Biomedical Engineering, University of Strathclyde, Glasgow, UK

**Keywords:** haemoconcentration, autotransfusion, packed cell volume, haematocrit, light sensor

## Abstract

**Background:**

HemoSep^®^ is a commercial ultrafiltration and haemoconcentration device for the concentration of residual bypass blood following surgery. This technology is capable of reducing blood loss in cardiac and other types of “clean site” procedures, including paediatric surgery. Clinical feedback suggested that the device would be enhanced by including a sensor technology capable of discerning the concentration level of the processed blood product. We sought to develop a novel sensor that can, using light absorption, give an accurate estimate of packed cell volume (PCV).

**Materials and methods:**

A sensor-housing unit was 3D printed and the factors influencing the sensor’s effectiveness – supply voltage, sensitivity and emitter intensity - were optimised. We developed a smart system, using comparator circuitry capable of visually informing the user when adequate PCV levels (⩾35%) are attained by HemoSep^®^ blood processing, which ultimately indicates that the blood is ready for autotransfusion.

**Results:**

Our data demonstrated that the device was capable of identifying blood concentration at and beyond the 35% PCV level. The device was found to be 100% accurate at identifying concentration levels of 35% from a starting level of 20%.

**Discussion:**

The sensory capability was integrated into HemoSep’s^®^ current device and is designed to enhance the user’s clinical experience and to optimise the benefits of HemoSep^®^ therapy. The present study focused on laboratory studies using bovine blood. Further studies are now planned in the clinical setting to confirm the efficacy of the device.

## Introduction

Following cardiac surgery, blood loss and allogeneic blood transfusions are associated with increased mortality and morbidity.^1–4^ Allogeneic blood transfusions have a number of risks, including febrile and allergic transfusion reactions^5^ and an increased likelihood of severe postoperative infections.^6^ Although transmission of infections such as hepatitis B/C and HIV have been virtually eliminated in first world countries through blood transfusions,^7^ inadequate blood screening in developing countries has failed to mitigate this considerable risk. Additionally, significant blood transfusion costs arise from the need to acquire, process, store and administer donated blood products.^8^ Autotransfusion technologies have offered an alternative to transfusion and they have been used to salvage and re-transfuse the patient’s own blood. Such blood concentration (haemoconcentration) techniques have included modified dialysis and centrifugation technologies to concentrate cell populations and remove excess water and plasma^9–11^ and these are relatively complex to use and require specialist technical knowledge to operate.^12^ Haemodilution strategies during cardiopulmonary bypass (CPB) procedures have adverse effects on fluid balance^13^ and the significant decrease in patient packed cell volume (PCV) and haemoglobin is associated with perioperative morbidity and mortality^14^ and increased blood loss.^15^

HemoSep^®^, a technology developed at the University of Strathclyde and manufactured and marketed by Brightwake Ltd (Nottingham, UK) is an ultrafiltration and haemoconcentration device for the concentration of residual bypass blood following surgery. This technology is capable of reducing blood loss in cardiac and other types of “clean site” procedures, including paediatric surgery. HemoSep’s^®^ blood bag component uses a membrane-controlled superabsorber-driven plasma-removal process which can preserve all cell species (red blood cells, white blood cells, platelets, proteins and clotting residuals) and remove contaminants prior to re-transfusion. Its control membrane has a pore size that prevents migration of cellular species from the blood to the superabsorber, whilst allowing free passage of diluted plasma into the superabsorber. As a result, once haemoconcentration to a clinically acceptable level (a PCV in excess of 35%) is achieved, the concentrated blood product is transferred to a transfer bag for subsequent autotransfusion. Effective haemoconcentration is supplemented by placing the HemoSep^®^ bag on HemoSep’s^®^ orbital shaker to agitate the device to encourage movement of the cells across the membrane surface and discourage settlement within the pore structure. HemoSep^®^ efficacy has been demonstrated clinically using residual diluted bypass and circuit blood^12^ and its deployment was also associated with reduced postoperative bleeding and red blood cell transfusion, as well as a decrease in post-cardiac bypass inflammatory response.^16^

Having launched the HemoSep^®^ device into international markets, clinical feedback suggested that the device would be enhanced by including a sensor technology capable of discerning the concentration level of the processed blood product. PCV values provide key clinical decision-making data by determining when the HemoSep^®^ process should be stopped and the blood transfused back to the patient. Currently, the only way to measure PCV with any accuracy in processed blood is to use a manual method, which requires blood samples to be taken intermittently from the HemoSep^®^ reservoir for time-consuming processing and manual estimation using hospital laboratory services. Developing a more efficient method that will not negatively impact upon blood processing time is required to solve this problem. The on-line real-time measurement of blood cell concentration is a complex issue and is impacted by a number of factors and there are no devices on the market at present that are capable of making accurate PCV measurements under the dynamic conditions represented by the HemoSep^®^ device. HemoSep’s^®^ effectiveness and impact would be greatly improved by giving the clinician the ability to quantify the concentrated cell products within the blood at the point of care while a surgical procedure is ongoing rather than relying on delivering blood samples to a clinical laboratory and waiting a significant delayed period of time to obtain blood chemistry test results. Various photometry techniques have been used to calculate haematocrit, such as dual-wavelength near infrared (IR)-photoplethysmography,^17^ optical coherence tomography,^18^ Raman spectroscopy^19^ and spectral domain low coherence interferometry,^20^ which derive the quantitative measurement of the reflection or transmission properties of blood as a function of wavelength. Automated commercial technologies use absorbance spectrophotometry light transmittance through blood to calculate the haematocrit, in which the absorbance of light by the haemoglobin complex in blood allows for the derivation of haematocrit via predefined algorithms.

## Aims

We aimed to develop a light sensor system – interfacing with HemoSep’s^®^ current system – that is sensitive to the range of blood concentration levels seen following haemodilution strategies associated with CPB deployment. A novel absorbance spectrophotometry application is capable of providing on-line, real-time PCV measurements during HemoSep^®^ blood processing. By configuring and setting an electronic comparator system’s reference voltage that corresponds to a clinically relevant threshold PCV using manual PCV measurements, we sought to develop a light-emitting sensor that can give an accurate and immediate estimate of PCV. This smart system would be capable of visually informing the user when adequate PCV levels (⩾35%) are attained by HemoSep^®^ blood processing (thus, indicating that the blood is ready for autotransfusion). This involved;

- Development of a 3D-printed sensor housing for integration with the existing HemoSep^®^ bag technology.
- Optimisation of the sensing components to maximize sensitivity to discrete and relevant PCV changes, with a focus on voltage supply, resistor values and emitter intensity.
- Utilisation of a comparator circuit to provide visual feedback that informs the user when blood haemoconcentration is complete.

## Methods

### Blood preparation and PCV analysis

Fresh bovine blood was collected from a local abattoir in a sealed container and treated with 10,000 IUs of heparin sodium from porcine mucosa (Sigma Aldrich, Dorset, UK) in 50ml of 0.9% NaCl. To measure the PCV, blood samples were collected in capillary tubes by capillary action and one end was sealed with an inert wax. Tubes were spun in a Hawksley 127 haematocrit centrifuge (Hawksley and Sons Ltd., Brighton, UK) for 2 minutes and the PCV was read using a Hawksley micro-haematocrit reader. To haemodilute the blood samples to the appropriate PCV concentrations, varying volumes of 0.9% sodium chloride solution was added to the blood and the PCV was measured. Depending on the measured PCV, additional blood or saline was added to acquire the desired PCV for sensor assessment. Fifty millilitres of haemodiluted blood was collected and 1 ml blood samples were placed in absorption cell cuvettes with a 5 mm light path for spectral analysis (Hellma Analytics, Mullheim, Germany).

### Sensing components

Photometry components sensitive to blood haemoglobin (High Power Infrared Emitter (850nm), RS Components, Corby, UK and a photodetector (a monolithic photodiode with on-chip trans-impedance amplifier as a light-to-voltage device for wavelengths in the range of 300 nm to 1100 nm, RS Components)) were selected. The emitter’s voltage output is the product of the photodiode current times the feedback resistor and the internal feedback resistor is laser trimmed to 1 megaohm (MΩ).

### Development of a 3D-printed sensor housing unit

The sensor housing unit design was drawn using 3D computer-aided design (CAD) software (PTC Creo Suite, PTC Inc., Needham, MA, USA). A clip-shaped prototype was developed to enable a suitable interface between the sensor components and the blood samples and to allow for integration with the pre-existing HemoSep^®^ bag. In this proof-of-concept series of experiments, the diagnostic sensor was assessed in a static environment, using absorption cell cuvettes that mimicked the dimensions and blood volume that we would expect to measure under dynamic clinical HemoSep^®^ blood-processing conditions. Imminent clinical trials will allow us to incorporate the sensing technology into the hardware of the commercial HemoSep^®^ system as an adjunct to the core technology in a dynamic setting. The housing unit for the sensing technology was 3D printed using an EnvisionTEC^®^ Perfactory^®^ Desktop XL printer (EnvisionTEC GmbH, Gladbeck, Germany) with Magics^®^ software (Materialise, Sheffield, UK). The resolution selected for printing was 25 microns and parts were printed using HTM140 material, a methacrylic-/acrylic-resin manufactured by EnvisionTEC^®^ with ABS-like material properties. The clip was designed to allow a distance of 8 mm between the emitter and the detector. This was a suitable distance that enabled the HemoSep^®^ bag to securely attach to the clip and it allowed for optimisation of the sensing components during experimental work by ensuring the absorption cell cuvette slots into the clip for blood PCV analysis.

### Optimisation of the sensing components

#### Supply voltage

The sensing technology must be capable of detecting and delineating the PCV values through the clinically relevant blood range (5-40%). Since the sensor converts light readings to a voltage - and voltage is proportional to blood concentration - a calibration curve of blood concentrations against voltage was generated to profile the voltage response and to select the optimal supply voltage for the technology. Using a DC power supply (Digimess PM3006-2, Digimess Instruments Ltd., Derby, UK), calibration curves of PCV against voltage response for three voltage outputs (5V, 10V and 15V) were generated. PCV values in 5% increments from 5-40% were assessed using 1 ml blood samples placed in cuvettes in the sensor housing unit and the voltage response was acquired via a USB analog input channel of a LabView data acquisition (DAQ) module (NI USB-6225, National Instruments, Austin, TX, USA) and viewed with NI LabVIEW 2013 (National Instruments).

#### External resistor values

The optimal sensitivity for the sensing components was defined by assessing the sensor response with a series of fixed resistors. Resistors were used to assess how the change in sensitivity would affect the linearity of the response and the sensor’s ability to delineate discrete PCV changes. The gradient of the curve was assessed to confirm if this configuration was capable of delineating voltage responses through the whole 5-45% PCV range and a linear trend-line determined optimal resistor selection. Twelve external resistor values were selected between 47.84 kiloohms (kΩ) and 720 kΩ. Based on the profiles generated from the 12 resistors, resistors with higher values (between 470kΩ and 1000kΩ) were assessed at PCV values between 30% and 45% to focus on resistors sensitive enough to delineate voltage responses within this crucial PCV range. For the external resistor measurements, a total of 17 resistor values were assessed, ranging from 47.84 kΩ to 1000 kΩ. For the lower resistor values (47.84 to 720 kΩ), nine PCV values ranging from 5% to 45% were assessed using an average of three blood samples taken per PCV value (324 blood samples). For the higher resistor values (470 to 1000 kΩ), five PCV values ranging from 30% to 43% were assessed using an average of three blood samples taken per PCV value (75 blood samples). There was no statistical difference between the three samples averaged. All blood samples were gently agitated before placement in the sensor to prevent settling of the cells in the cuvette and each sample was measured immediately and the data plotted. The optimal resistor was then selected for inclusion into the sensor circuitry and used for subsequent experimental procedures.

#### Emitter intensity

It was vital to standardise the emitter intensity following the assessment of its influence on sensor responsivity. Emitter current values were tested at 100 milliamps (mA) and 133mA. To calculate the resistance required for these current values, the following equation was used:

$$R=\left(\frac{Vsupply-Vf)}{i}\right)$$

Where V_*supply*_ is 5V, V_*f*_ for the emitter is 3V (in accordance with the emitter’s datasheet value) and *i* is the desired current. From this, each current’s resistance value was:

$$\begin{array}{l} 100mA:\;R\;=\;19.5\Omega \\ 133mA:\;R=\;15\Omega \end{array}$$

Profiles of the voltage response for PCV values between 30-45% were assessed using the two selected emitter intensities.

### Developing a smart system - comparator and light-emitting diode (LED) circuit

Following optimisation of all parameters that control the sensitivity and emissivity of the sensor technology, a comparator circuit was designed and built to provide visual response feedback to the end-user. The comparator system used one green and one red light-emitting diode (LED) and its premise was that illumination of the red LED would be the default configuration when the blood is not adequately haemoconcentrated (PCV<35%). Experimental work to generate the voltage values was carried out to select a reference voltage that corresponds to the PCV that we want the blood to reach before giving it back to the patient. When a reference voltage is reached, the red LED will switch off and the green LED illuminate to ultimately inform the user when the haemoconcentration is complete. We aimed to set the comparator’s voltage reference to correspond to a PCV of greater than 35%. The reference was defined by generating voltage readings for the range of blood concentrations between 30 and 42%. One millilitre blood samples in cuvettes were placed in the sensor and the voltage value for each PCV concentration (30%, 35%, 38%, 40% and 42%) was measured using the LabView DAQ module. Graphs of voltage response against time were generated and the reference voltage value for the comparator system that corresponded to the PCV greater than 35% was selected.

## Results

### Development of a 3D-printed sensor housing unit

**Figure 1.**
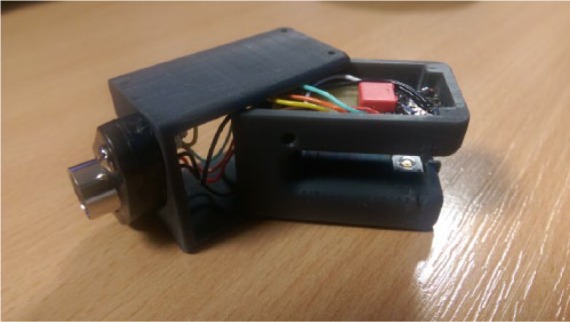
3D-printed clip built in two parts to house the electronic components.

The sensor housing unit was 3D printed in a matte black material to eliminate light scattering. It was developed in two parts to allow the electronic components to be housed within the unit (Figure 1). A standard 7-way DIN connector was connected to enable attachment to a power supply.

### Optimisation of the sensing components

#### Supply voltage

**Figure 2.**
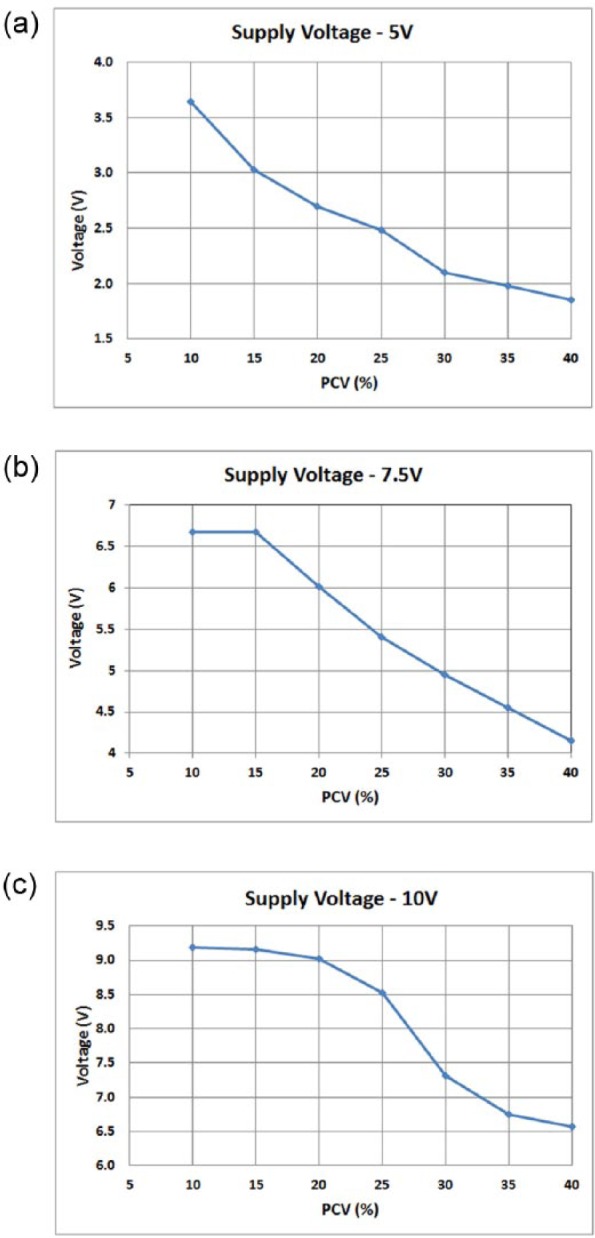
Sensor voltage responses for the selected blood concentration (PCV) values using a (a) 5V, (b) 7.5V and (c) 10V power supply.

From the analysis of the three selected supply voltages, the 5V supply voltage displayed a clear linear response and a marked delineation in voltage readings throughout the whole PCV range (Figure 2). The 7.5V and 10V supply voltages had a saturated voltage response in the more diluted PCV values (between 10 and 20%) and were, thus, incapable of differentiating between these values. For this reason, the 5V supply voltage was selected for subsequent experimental work on this sensor technology.

#### External resistor values

**Figure 3.**
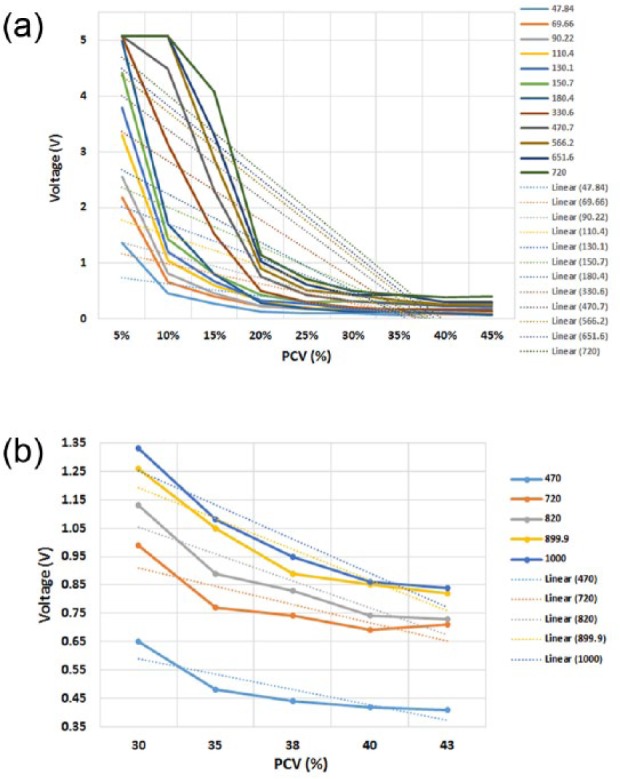
Voltage response of the sensor using various external resistors: a) 12 external resistors between 47.84kΩ and 720kΩ at the 5-45% PCV range and b) 5 higher value external resistors (between 470 kΩ and 1000 kΩ) at the 30-43% range. Linear trend-lines were added to ascertain the steepest gradient. The highest resistor values tested displayed the greatest voltage delineation at these PCV values.

All external resistors measured showed a marked delineation in voltage responses in low PCV values (5-25%) (Figure 3a). However, steeper gradients were observed using the higher resistor values of 330.6KΩ and above. All resistors were incapable of providing variable voltage responses at PCV values of 25-45%. Higher resistor values were then tested on PCV samples between 30-43% to identify optimal resistor values for higher blood concentrations (Figure 3b) and it was observed that the highest resistor values tested displayed the greatest voltage delineation at these PCV values. The 1000 KΩ resistor showed the most sensitive response at this PCV range and this was selected for the sensor technology.

#### Emitter intensity

**Figure 4.**
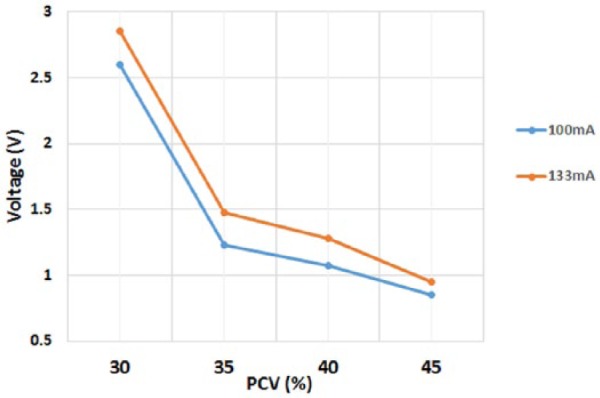
The effect of emitter current on the voltage response using PCV values between 30-45%.

For PCV values between 30-45%, the influence of emitter intensity was analysed for the 100mA and the 133mA values (Figure 4). The acquired signal had a near linear response with both current values. As expected, the higher current displayed the greater voltage response, so the 15Ω resistor (133 mA current) was incorporated into the sensing technology.

### Developing a smart system - comparator and LED circuit

**Figure 5.**
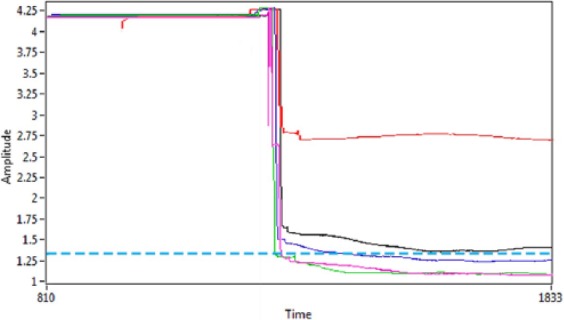
Voltage response acquired when placing varying blood concentration samples in the sensing circuit. The 4.23V baseline amplitude decreased to a greater extent as the blood concentration of the samples increased. The comparator reference voltage was set at 1.35V which corresponded to a PCV greater than 35%.

Using the 5V power supply, the baseline voltage response was 4.23V (Figure 5). Reduction in amplitude from this baseline corresponded to placement of the blood sample in the sensing technology. As the PCV increased, the voltage response decreased and it was possible to show a measureable difference between the voltage values at these blood concentrations. The reference value for this circuit was set at 1.35V (shown as a dashed line in Figure 5) which indicated that the blood was greater than 35%. When the concentration reached this voltage value, the comparator circuit switched the LED from red to green illumination immediately and this was capable of informing the user that haemoconcentration was complete.

### The completed sensor prototype

**Figure 6.**
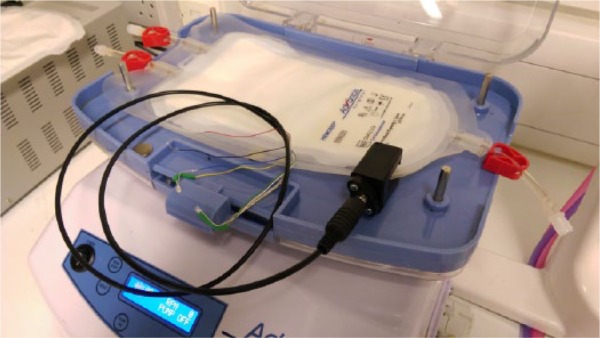
Final configuration of the sensor clip prototype integrating with HemoSep’s^®^ pre-existing technology. The current sensor can be connected to a standard 5V power supply via the red and black leads and the red and green LEDs for user visual feedback are connected to the clip via a cable.

Figure 6 displays the configuration of the sensor prototype integrated with HemoSep’s^®^ existing technology.

## Discussion

The completed sensor technology works effectively for a PCV reference value of >35% and it is consistent through a range of blood batches and samples and, since the clip is externally fixed, it does not interfere with the activity of the HemoSep^®^ concentration bag. The prototype is currently designed for simple connection to a power supply (shown by the black and red leads on the black cable). As a standard 5V power supply is used for the sensor, future iterations of this technology will enable the clip to connect and be powered through HemoSep’s^®^ orbital shaker. The green and red LEDs that provide the visual user feedback will also be integrated into HemoSep’s^®^ shaker.

The world market for cell salvage is significant. Pioneered in open-heart surgery, it is now practiced in orthopaedic and trauma surgery, transplantation and renal and hepatic surgeries. The UK has circa 50,000 open-heart surgical procedures annually and 50% use cell recovery. There is an increased recognition of the cost and risk associated with the use of donor blood/blood components so the cell harvesting market is expected to grow. It is anticipated that incorporating sensory devices into the HemoSep^®^ blood bag will enable significant enhancement of the device, both in ease of use and speed of detection, for the end-user and will enable a more rapid uptake of this technology in clinical practice with a novel blood concentration technology aimed for use at the point of care. The current device requires blood samples to be taken during surgery and sent to the laboratory for testing cell concentration. This time-consuming step will be eliminated and will enable the equipment to be used in a larger variety of applications and open up new markets, for example, in the field for military/emergency procedures. This, also, will create opportunities for re-designing and extending the product range and potential use for portable equipment. This study developed and integrated a detection sensor into the product to enable accurate data outputs at the point-of-care to facilitate surgical decisions in situations where there are high levels of blood loss and transfusion using the patient’s own “recycled” blood is required. The detection capability is able to be incorporated into the hardware of the HemoSep^®^ device and it is a durable, multi-use adjunct to the core technology. This sensor technology will considerably enhance the user experience, enabling clinicians to clearly define the end point of the haemoconcentration process prior to autotransfusion.
